# Electromyographic Study for the Objective Evaluation of Glare-Induced Discomfort in Patients With Keratoconus

**DOI:** 10.7759/cureus.80466

**Published:** 2025-03-12

**Authors:** Argyro D Plaka, Sotirios Plainis, Petros Chatzakis, Georgios Kontadakis, George Markakis, George Kymionis, Ioannis Pallikaris, Haralampos Siganos

**Affiliations:** 1 Institute of Vision and Optics, Medical School, University of Crete, Heraklion, GRC; 2 First Department of Ophthalmology, "G. Gennimatas" Hospital, National and Kapodistrian University of Athens, Athens, GRC

**Keywords:** bitalino (r)evolution kit, de boer scale, discomfort glare, electromyography, keratoconus

## Abstract

Purpose: To use a portable electromyographic (EMG) system for the objective evaluation of glare-induced discomfort in patients with keratoconus.

Methods: Fifty-five eyes of 31 patients and 24 controls were enrolled in this retrospective case-control study. The electrical activity of the mimic muscles was recorded at three different luminances (500, 3000, and 6000 lux) using a portable EMG system. A discomfort glare value was obtained as the signal/noise ratio for each recording. All the patients were asked to grade their discomfort after exposure to each luminance using the subjective de Boer scale. Corneal topography-based wavefront analysis, pupil size, and angle kappa were calculated to identify possible significant correlations.

Results: Mean values of discomfort glare index (signal/noise ratio) for the patients’ group were 1.66 ± 1.5 at 500 lux, 2.71 ± 1.68 at 3000 lux, and 3.16 ± 1.92 at 6000 lux; for the control group, 1.4 ± 0.4 at 500 lux, 1.9 ± 0.86 at 3000 lux, and 2.13 ± 0.9 at 6000 lux. There was a statistically significant difference at the level of 0.05 between the two groups only at the 6000-lux illuminance level. There was a significant Spearman’s correlation at all luminances between the objective electromyographic recording and the subjective grading of the discomfort feeling. There were no statistically significant correlations between electromyographic responses and keratometric values, pupil size, or angle kappa. Trefoil and coma were significantly correlated with discomfort glare-estimated electromyographic values.

Conclusions: Patients with keratoconus experience higher levels of discomfort when exposed to a glare source, as measured objectively with electromyographic recordings, which may affect their quality of vision.

## Introduction

Keratoconus is a corneal ectatic disorder in which the cornea takes on a conical shape due to significant thinning and protrusion. It is no longer considered a non-inflammatory condition due to the discovery of proinflammatory cytokines involved in its etiopathogenesis [1]. The initial clinical signs of the disease are unstable refraction and irregular astigmatism, and progression is usually expected until the third decade of life. The main diagnostic finding in corneal topography is inferior corneal steepening accompanied by central corneal thinning. Although corneal crosslinking is the treatment of choice for halting the disease [2], corneal transplantation is necessary when the disease is advanced in its late stages [3].

Visual correction in patients with keratoconus and irregular cornea, who mainly exhibit myopia and astigmatism from the early years of disease onset, is usually achieved by using glasses or contact lenses. Despite their sphero-cylindrical correction, patients with keratoconus habitually experience multiple ghost images and halos and suffer from increased retinal straylight due to higher amounts of corneal higher-order aberrations. Retinal straylight is further exaggerated under conditions of excessive brightness (i.e., the headlights of oncoming vehicles), resulting in varying degrees of discomfort, from a mild sensation to an intolerable feeling of pain [4, 5].

Discomfort glare is defined as ocular stress that does not necessarily impair the visibility of an object [6]. It describes the response to bright light under dim- or dark-adapted conditions accompanied by a strong spasm of the extraocular muscles. While the visual system responds well to a wide range of illuminations through adaptation mechanisms, such as changes in pupil diameter, if light levels are too high, adaptation mechanisms are saturated, and the bright regions of the scene may lead to discomfort [6]. Discomfort glare is usually evaluated subjectively using grading scales, such as the de Boer scale, which spans from one to nine, where nine corresponds to just noticeable discomfort and one to unbearable discomfort [7]. Although the de Boer scale is the most frequently used subjective method for discomfort glare evaluation, its usefulness remains controversial [8].

According to Jinabhai et al. [9], intraocular forward light scatter, which is related to disability glare and contrast loss, is significantly greater in patients with keratoconus than in normal subjects. The high values in these patients may be related to structural degradation of the cornea rather than to age or scarring. Statistically significant elevations in retinal straylight have been reported after corneal crosslinking in several studies; however, there are no published data regarding discomfort glare in keratoconic eyes. In contrast to disability glare, which causes a reduction in visibility, discomfort glare is defined as the masking effect caused by light scattered in the ocular media, which produces a veiling luminance over the field of view and results in reduced visual performance [5,9]. The impact of discomfort glare on patients with keratoconus and corneal irregularities has not yet been studied.

There have been several previously published attempts to objectively measure discomfort glare [10, 11], while strong binocular summation effects have been reported [10]. These studies have linked the electromyographic (EMG) activity of the orbicularis oculi and other nearby muscles, i.e., corrugator supercilii, to discomfort glare. They showed that the amplitude of EMG in response to a light stimulus of a particular intensity was closely related to subjective responses, providing an objective estimate of the severity of the response to bright light. Similarly, Berman et al. [11] found that increasing objective measures and subjective discomfort are associated with increased glare luminance.

The current study aims to evaluate discomfort glare subjectively and objectively using EMG recordings in patients with keratoconus.

## Materials and methods

Population

Fifty-five dominant eyes of 31 patients and 24 controls (13 women and 18 men in the patient group vs. 10 women and 14 men in the control group) aged 18-45 years old were enrolled in this retrospective case-control study. All participants visited the outpatient service of the Institute of Vision and Optics, School of Medicine, University of Crete in Heraklion, GRC, either for their regular or postoperative follow-up (patient group) or for a routine ophthalmic examination (control group). Written consent was obtained from all participants prior to enrollment in the study. This study conformed to the tenets of the Declaration of Helsinki and followed a protocol approved by the University of Crete Research Board (decision number: 1/13-02-2012).

The inclusion criteria for the patients’ group were non-progressive keratoconus or progressive keratoconus at least one year after corneal crosslinking. This time period was selected to avoid measuring the discomfort glare affected by the early postoperative period. All patients were graded as stage 1 or 2 keratoconus according to the adapted Amsler-Krumeich classification (mean central keratometry <53 D and minimum corneal thickness >400 μm) [12]. An age-matched group served as the control group.

The exclusion criteria were a history of multiple corneal surgeries, previous cataract or retinal surgery, best-corrected visual acuity less than 0.20 logMAR, refractive errors higher than 8.00D, and the presence of any other ocular or systemic disease, such as superficial keratitis and/or allergic conjunctivitis that could affect the results. 

Methods 

Electromyographic-based objective responses were obtained using a portable and easy-to-use biomedical engineering platform, the BITalino (r)evolution kit (PLUX Biosignals, Lisbon, Portugal), which has been used in electrocardiography [13] and electroencephalography [14] studies, showing high correlation with classic EMG systems [15]. Electrical activity of the extra-ocular muscles was recorded with three small surface electrodes (positive, negative, and ground). Prior to the beginning of the protocol, EMG-based responses from the BITalino system and a neurophysiological platform CED 1902 (CED Electronics, Cambridge, United Kingdom) were compared to determine the optimal position of the electrodes around the eye. Both the BITalino system and CED 1902 platform have a sampling rate of 1000 Hz. 

The examination was performed in a dark room with the patient’s head stabilized on a chinrest. The light source was positioned and centered at a distance of approximately 60 cm. Stimulus luminance was moderated with filters placed in front of the fixed 100 W diffuse white (polychromatic) light source. Two 9 mm silver-silver chloride (Ag/AgCl) surface electrodes were attached to the skin periocularly, placed below the lower eyelid (positive) and above the nasal part of the eyebrow (negative) of the examined eye. The third electrode was positioned behind the ear and served as ground. Before the electrode application, the skin was cleaned with alcohol. The electrodes were attached with conductive paste, and the electrode impedance was maintained below 6 kW at all times. The dominant eye was used with a natural pupil and optimal refractive correction for far. 

The EMG signals from the electrodes were fed into an amplifier, a narrow band filter, and a separate receiver unit. Signals were filtered with a narrow band (20 Hz at half height) time-active filter tuned to 180-200 Hz. The stimulus strength of the light source was determined in terms of the vertical illuminance (lx) at the level of the cornea. Before stimulus presentation, the spontaneous EMG activity of the subject relaxed, and with eyes open, was recorded to verify the baseline activity of the periocular muscles. The EMG activity was recorded for a period of four seconds after the onset of the glare stimulus. Three recordings were performed at each luminance (500, 3000, and 6000 lux) with a recovery period of at least one minute between successive trials to desaturate the photoreceptors from light exposure. In addition, at the end of exposure to each luminance, subjective responses were assessed, with the patient grading the level of discomfort on the de Boer scale, from one to nine, where nine corresponds to minimal discomfort and one to maximal discomfort.

Following the examination, a topography-based wavefront analysis of the cornea was performed under the same room conditions, and pupil size and angle kappa were calculated using a wavefront aberrometer (I-trace, Tracey Technologies, Houston, Texas, USA). The angle kappa is defined as the angle between the visual axis (the line connecting the fixation point with the fovea) and the pupillary axis (the line that perpendicularly passes through the entrance pupil and the center of the curvature of the cornea). High-order aberrations are optical irregularities of the refractive media that cannot be corrected with glasses or contact lenses and may cause halos, glare, starbursts, etc. They are measured by root mean square (RMS) values in micrometers based on Zernike polynomials. It is well known that patients with keratoconus suffer from increased high-order aberrations when compared to normal subjects; in particular, coma and third-order RMS may be useful in the diagnosis of a topographical classification of keratoconus [16]. 

The total examination time was approximately 20 minutes.

Data analysis

The EMG recordings were saved to a software (Opensignals, PLUX biosignals) through a Bluetooth connection and then transferred for further analysis (OriginPro, OriginLab Corporation, Northampton, MA, USA). The data are presented in a two-dimensional initial amplitude-time plot, and the blinks are marked by large fluctuations in the amplitude of the waveform. During further data processing, the absolute values of the amplitude measurements were used on the vertical axis, and a Fourier transformation (FFT) converted the time to a frequency on the horizontal axis to obtain the corrected amplitude-frequency plot. An area below 180-200 Hz was used for the discomfort glare evaluation. At these frequencies, the effect of flickering in the analysis was eliminated, and the ratio of signal to noise (signal/noise), which was used as an objective index of discomfort glare, obtained the optimal value. 

All four recordings (noise at 0 lux and electromyographic activity at 500, 3000, and 6000 lx) were collected using an Excel spreadsheet (Microsoft Corp., Redmond, WA, USA). The mean value from the three measurements performed for each amount of corneal illuminance (500 lux, 3000 lux, and 6000 lux) was divided by the mean value in the absence of the glare source (noise at 0 lux) to obtain the signal/noise ratio, which is a discomfort glare index for each corneal illuminance level. 

Statistical analysis

Stata software (version 20.0; StataCorp LLC, College Station, TX, USA) was used for statistical analysis of the results. Continuous variables are presented as mean ± standard deviation. A sample size analysis (G*Power 3.1.3, Franz Paul, Universität Kiel, Germany) was performed for independent samples, a large difference effect size, a statistical power of 80%, and a statistical significance level of 0.05, resulting in 24 patients participating in each group in this retrospective study. The normality of the distribution of the discomfort glare index, mean k, and I-trace data regarding topography-guided wavefront analysis, pupil size, and angle kappa were checked for the patients and the control group. Shapiro-Wilk’s test and visual inspection of their histograms, normal Q-Q plots, and box plots showed that the majority of the parameters were not normally distributed. Therefore, non-parametric tests (i.e., the independent samples Mann-Whitney U Test or the independent samples Kruskal-Wallis Test) appropriate for each comparison were performed to analyze the data. Statistical significance was set at p-value <0.05. Continuous variables are presented as mean ± standard deviation.

Since both the distributions of EMG recordings and de Boer scale assessment were not normally distributed, Spearman’s rank correlation coefficient was calculated to determine correlations between EMG data and subjective discomfort scores. Spearman’s ρ is appropriate for both continuous and discrete ordinal variables and indicates the direction of association between the two variables.

## Results

Prior to the beginning of the study, EMG responses from 11 normal subjects (aged between 23 and 35 years old) were compared between the BITalino system and the standard neurophysiological amplifier CED 1902 to find the optimal placement of the electrodes around the eye. Correlation between the recordings of the two systems was high (p<0.001) when the electrodes were placed in the aforementioned arrangement: the two electrodes were placed below the lower eyelid (positive) and above the nasal part of the eyebrow (negative) of the examined eye while the third electrode was positioned behind the ear and served as ground.

Fifty-five dominant eyes of 31 patients with keratoconus and 24 controls aged 18-45 years participated in the study. The right eye was examined in 30/55. The patient group included 25 patients with keratoconus at least one year after corneal crosslinking and six patients with non-progressive keratoconus.

Mean ± SD values of the discomfort glare index (signal/noise ratio of EMG responses) for the patients’ group were 1.66 ± 1.5 at 500 lux, 2.71 ± 1.68 at 3000 lux, and 3.16 ± 1.92 at 6000 lux with corresponding values of 1.4 ± 0.4, 1.9 ± 0.86, and 2.13 ± 0.9 for the control group. A statistically significant difference between the two groups was found at 6000-lux corneal illuminance (p=0.045). The difference was marginally statistically insignificant at 3000 lux (p=0.062). Regarding the subjective responses, the mean ± SD values of de Boer scaling were for the patients group 8.5 ± 0.3 at 500 lux, 5.7 ± 0.4 at 3000 lux, and 4.1 ± 0.4 at 6000 lux with corresponding values of 8.9 ± 0.2, 6.6 ± 0.3 and 5 ± 0.4 for the control group. There were no statistically significant differences between the two groups (p-value > 0.05 in all comparisons with the independent samples Mann-Whitney U Test) (Table 1).

**Table 1:** Objective (electromyographic response ratio) and subjective (scaling) values of discomfort glare for the range of corneal illuminances tested The mean value of the discomfort glare index (signal/noise ratio of EMG responses) ± standard deviation is presented in patients' and controls' cells. An independent samples Mann-Whitney U test was performed, and a p-value < 0.05 was considered statistically significant (pointed with *). EMG: electromyographic

Evaluation	Luminance (lux)	Patients	Controls	p-value
Objective	500	1.66 ± 1.5	1.40 ± 0.4	0.812
	3000	2.71 ± 1.68	1.90 ± 0.86	0.062
	6000	3.16 ± 1.92	2.13 ± 0.90	0.045*
Subjective	500	8.5 ± 0.3	8.9 ± 0.2	0.899
	3000	5.7 ± 0.4	6.6 ± 0.3	0.125
	6000	4.1 ± 0.4	5 ± 0.4	0.138

**Figure 1:**
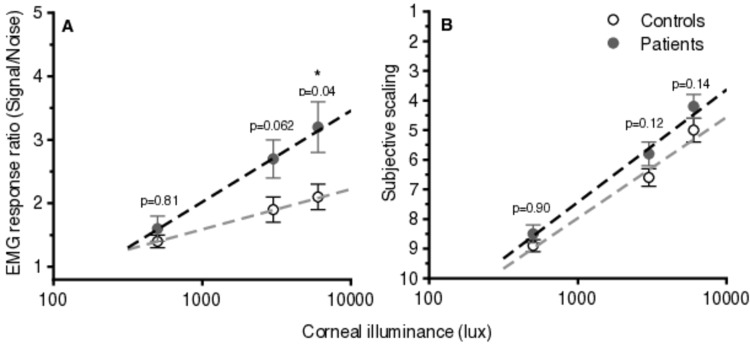
Plots of average (± standard error) objective values of discomfort glare (EMG responses) (A) and subjective scaling (B) as a function of corneal illuminance in a logarithmic scale for the two groups (patients vs. controls). A linear relationship between objective and subjective responses to discomfort glare with the logarithm of corneal illuminances is found. The dashed lines form linear regression plots (r-squared > 0.97 in all cases, thus suggesting a strong relationship between the variables). The y-axis plots the EMG response ratio which has no units, and the x-axis plots corneal illuminance in a logarithmic axis. P-values correspond to the difference between the two groups at each corneal illuminance level (a p-value < 0.05 was considered statistically significant). EMG: electromyographic

Figure 1 plots the mean value of objective (EMG response signal/noise ratio) and subjective responses for the control and the patients’ groups, showing a linear correlation with the logarithm of corneal illuminance. 

**Figure 2:**
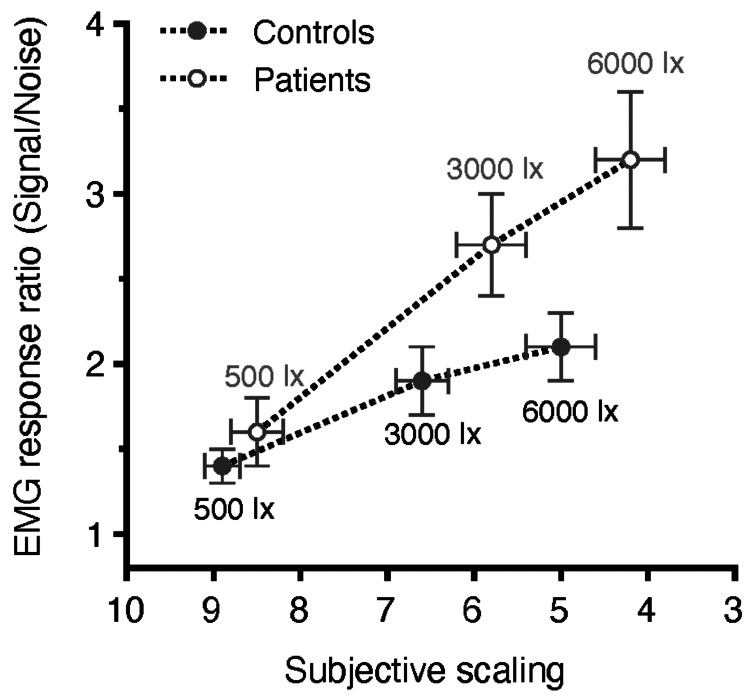
Correlations (± standard error) between the average values of discomfort glare (EMG responses) and subjective scaling at the three levels of corneal illuminance for the two groups (patients versus controls). The y-axis plots the EMG response ratio which has no units; the x-axis plots subjective scaling which has no units. Note that in subjective scaling, nine corresponds to minimal and one to maximal discomfort. Corresponding p-values are depicted in Table 1. EMG: electromyographic

Spearman's ρ was calculated to search for correlations between the objective EMG response ratio and subjective scaling of the discomfort feeling. The overall Spearman’s ρ value for both groups at all illuminances was -0.79 (p<0.001). More specifically, the Spearman’s ρ value was for the patients’ group -0.58 (p<0.001) at the 500-lux illuminance exposure, -0.86 (p<0.001) at the 3000 lux exposure, and -0.88 (p<0.001) at the 6000 lux exposure, and for the control group -0.31 (p=0.07), -0.75 (p<0.001), and -0.77 (p<0.001), respectively. These results show that the objective value that arises from the EMG measurement ​​​​​corresponds well to the subjective scaling of discomfort using the de Boer scale for both groups (patients and controls). Figure 2 plots the correlation between the mean objective index (EMG response) and subjective rating of discomfort glare for both groups, showing a negative correlation, i.e., the higher the objective response, the higher the subjective rating of discomfort glare.

The mean pupil size was 5.1 ± 0.9 mm and 5.3 ± 0.9 mm for the control and patients’ groups, respectively. The mean angle kappa for the control group was 0.28 ± 0.15, and for the patients group, 0.34 ± 0.16. Pupil size and angle kappa are potential confounding factors, so they were included in our analysis. However, we did not find a statistically significant difference in pupil size or angle kappa between the groups of patients and controls. In addition, no difference between the two groups was found in their distributions. Moreover, pupil size or angle kappa were not significantly correlated to keratoconus or to discomfort glare index. Therefore, pupil size and angle kappa did not confound our study results. The mean keratometry value for the control group was 43.00 ± 2.28 D, and for the patients’ group was 44.25 ± 3.63 D. There was neither a statistically significant difference nor a significant correlation between pupil size, kappa angle, mean keratometry values, and the discomfort glare index in either group. 

Topography-based wavefront aberrations 4 mm from the central cornea were analyzed to identify possible significant correlations. For the control group, the mean RMS values of the total high-order aberrations, high-order astigmatism, spherical aberrations, and coma were 0.13 ± 0.06 μm, and the mean RMS values of the total high-order order astigmatism 0.06 ±0.05 micrometers, spherical aberration 0.07 ± 0.03 μm, coma 0.06 ± 0.05 μm and trefoil 0.07 ± 0.03 μm. For the patients’ group, the mean RMS value of total high-order aberrations was 0.80 ± 0.47 μm, high-order astigmatism 0.12 ±0.09 μm, spherical aberration 0.13 ± 0.09 μm, coma 0.65 ± 0.46 μm and trefoil 0.43 ± 0.22 μm. The differences in all high-order topography-based aberrations between the two groups were statistically significant (p<0.001, independent samples Kruskal-Wallis test), but no significant correlations were detected to link the high-order aberrations difference to the value of the discomfort glare index.

However, coma and trefoil were significantly correlated with the discomfort glare index, disregarding the participant's corneal status at all corneal illuminances. The Spearman’s ρ was for coma 0.296 (p=0.03) at 500 lux, 0.363 (p=0.007) at 3000 lux, and 0.326 (p=0.016) at 6000 lux, and for trefoil, 0.277 (p=0.04) at 500 lux, 0.317 (p=0.02) at 3000 lux, and 0.323 (p=0.017) at 6000 lux. The correlation was linearly positive, indicating that an increase in coma and trefoil was highly correlated with an increase in the discomfort glare index regardless of the corneal status of the subject.

## Discussion

Discomfort glare is a complex and multidisciplinary neurophysiological parameter that is difficult to quantify [17]. It is known that discomfort glare is accompanied by strong contractions or spasms in muscles surrounding the eye. Therefore, an objective method to estimate discomfort glare is to monitor the EMG activity of extraocular mimic muscles. The EMGs obtained from surface electrodes represent the sum of the action potentials in the underlying muscle fibers, and the peak in the amplitude reflects high-frequency motor neuron activity.

In our study, we showed a portable low-cost device system (the BITalino system), showing a good correlation in EMG response values with a standard neurophysiological amplifier (Pearson’s r=0.75, p<0.001). This is in agreement with previous studies [15]. Additionally, a high correlation was detected between participants’ EMG responses and subjective discomfort rating on the de Boer scale. The de Boer scale is currently the conventional way to estimate discomfort glare, but it is biased because of its nature [8].

It is well known that discomfort glare has a significant impact on everyday life activities. For example, eyestrain when performing tasks reliant on a digital environment can cause discomfort, affecting productivity and quality of life, and has an impact on the corneal surface [18]. In addition, the high prevalence of monitor display use may be negatively associated with reading performance [19]. Driving performance may also be seriously affected by uncomfortable glare conditions caused by car headlights or road lamps [20]. The effect of discomfort glare sensation [20] on driving performance is well established. 

To our knowledge, this is the first study to objectively evaluate the impact of discomfort glare in patients with keratoconus. Our results show increased average values of EMG responses in the patients vs. control groups at all corneal illuminances, but the difference was statistically significant only at 6000 lux and marginally insignificant at 3000 lux (Figure 2). This finding indicates that patients with irregular corneas may be compromised when facing straightforward light of up to 3000 lux illuminance under laboratory conditions, while exposure to a brighter source (6000 lux) seems to cause significant disturbance in the patient compared to the control group (Figure 1). Larger sample size and/or inclusion of patients with advanced keratoconus (stage III or IV of Amsler Krumeich classification) may have resulted in statistically significant differences also at a corneal illuminance of 3000 lux. These symptoms may be alleviated with the use of customized contact lenses or eyeglasses [22], which are known to improve everyday activities such as driving performance and comfortable screen use in patients with keratoconus. 

Several factors, such as pupil size [23] and eye movements [24], have been previously investigated in relation to discomfort glare. Other studies attempted to link discomfort glare with certain physiological indices such as pupil size fluctuations, particularly pupillary hippus, without significant results [25]. No significant correlation between pupil size or angle kappa with EMG-derived discomfort glare index was found in the analysis of the results, while no difference was observed in these parameters between the two groups. Additionally, the mean corneal curvature did not seem to correlate with discomfort glare. 

Topography-based high-order aberration values differed significantly between the two groups, which is not surprising since the patient group consisted of individuals with irregular corneas [26]. However, despite this difference, the discomfort glare index for the 6000 lux group (i.e., total, spherical aberration, coma, and trefoil) did not show a different correlation with high-order aberrations between the two groups. 

Therefore, the increased discomfort glare values in the patient group could not be attributed to the optical (corneal and/or pupil) parameters investigated in our study. Thus, neural adaptation mechanisms may also be involved [6]. It is well established that the state of retinal (photoreceptor) adaptation also influences discomfort glare sensation [27], i.e., when fully light-adapted, discomfort glare is mild, but in the dark or partially dark-adapted state, a strong glare response is evident, which may be accompanied by pain. Such effects are commonly experienced when driving at night when the headlamps of oncoming vehicles induce considerable discomfort and ocular stress [10]. In addition, neural adaptation mechanisms may also be involved, as evident by the strong binocular summation effects of discomfort glare [27]. Although neural filtering has been suggested in generic glare sensation models’ neurons [28], there are no studies showing any genuine effects of neural adaptation in patients with normal vision or with corneal disease.

Moreover, structural changes in the cornea, as already assumed for disability glare increase [9], may also be involved, resulting in higher levels of discomfort glare in the patient group. Additionally, the impact of the ocular surface on the discomfort glare index should be considered [18] because patients with irregular corneas have impaired corneal sensitivity and suffer from dry eye disease [29] or meibomian gland dysfunction [30]. Macular pigment optical density may also affect discomfort glare [31].

An interesting correlation between coma and trefoil and EMG responses was found at all illuminances, regardless of the corneal status of the subjects. This implies that coma and trefoil, which form higher-order aberrations of the anterior surface of the cornea, may play a role not only in visual quality but also in the causative mechanism of discomfort glare. Customized topography-guided ablation treatments and/or customized corneal crosslinking protocols could be developed using special laser-platform software for these patients to help them improve their quality of vision by reducing the amount of horizontal/vertical coma aberrations, which are predominant in irregular corneas, and other higher aberrations, i.e., trefoil. Further studies are required to confirm these findings.

Scientific interest in the quality and psychophysics of vision is rising. Discomfort glare is a parameter that cannot easily be measured but has an important role in the perception of vision. Discomfort glare objective assessment may offer patients with keratoconus customized contact lenses, spectacles, or intraocular lens designs that may control the amount of light entering the eye depending on environmental lighting conditions. Additionally, laser treatments in keratoconic corneas could be designed to target the management of high-order aberrations, especially trefoil and coma. The most important part is that every intervention may be assessed using this easy electromyographic technique described herein.

Limitations of the study have to do with the complex neurophysiological mechanism involved in discomfort glare formation. Therefore, electromyography may be a helpful but not the only tool in objectively evaluating discomfort. Of course, further studies are needed to completely understand the mechanisms involved in discomfort glare in patients with keratoconus.

## Conclusions

In conclusion, previous studies have investigated the effects of discomfort glare in healthy subjects and different age groups. The present study used a low-cost and reliable EMG system to evaluate objectively the level of discomfort under different lighting conditions in patients with keratoconus and resulted in a significant increase in discomfort glare index when exposed to corneal illuminance brighter than 3000 lux, which may affect their quality of vision. A potential role of the high-order aberrations, coma, and trefoil, in the intensity of discomfort glare has emerged and should be further investigated. This establishment, due to its portability, may be useful in developing and evaluating customized contact lenses or eyeglasses to help patients with keratoconus improve their everyday performance, especially under challenging circumstances, such as driving or screen exposure.
